# Analysis of Frailty Indices Based on Sociodemographic and Clinical Determinants in Older Women

**DOI:** 10.3390/healthcare13151791

**Published:** 2025-07-23

**Authors:** Filipe Rodrigues, Diogo Monteiro, Miguel Jacinto, Rui Matos, Nuno Amaro, Ricardo Pocinho, Sara Gordo, Sílvia Silva, Raul Antunes

**Affiliations:** 1 ESECS, Polytechnique of Leiria, 2411-901 Leiria, Portugal; filipe.rodrigues@ipleiria.pt (F.R.); diogo.monteiro@ipleiria.pt (D.M.); rui.matos@ipleiria.pt (R.M.); nuno.amaro@ipleiria.pt (N.A.); ricardo.pocinho@ipleiria.pt (R.P.); silvia.c.silva@ipleiria.pt (S.S.); raul.antunes@ipleiria.pt (R.A.); 2 Research Center in Sport, Health, and Human Development (CIDESD), 5000-558 Vila Real, Portugal; 3 CICS.NOVA.IPLeiria, 2411-901 Leiria, Portugal; 4 National Association of Social Gerontology, 3100-516 Pombal, Portugal; sara.gordo@ipleiria.pt; 5 School of Health Sciences, Polytechnic of Leiria, 2411-901 Leiria, Portugal; 6 Center for Innovative Care and Health Technology, 2411-901 Leiria, Portugal

**Keywords:** physical, frailty, older adults, community, health

## Abstract

**Background:** The aim of this study was to analyze levels of frailty, across physical, psychological, social, and overall dimensions, according to marital status, age, number of diagnosed illnesses, and number of medications taken in community-dwelling older women. **Methods:** The study included a total of 94 older women, aged between 60 and 89 years. All participants completed a sociodemographic and clinical questionnaire, as well as an instrument to measure physical, psychological, and social frailty, along with the total frailty score in the study participants. Group comparison test, such as the Kruskal–Wallis test, was applied. **Results:** Statistically significant differences (*p* < 0.05) in frailty were associated with marital status, clinical burden, and polypharmacy, with widowed/divorced individuals, those with more diagnosed diseases, and those taking more medications exhibiting higher physical and total frailty levels, while psychological frailty was notably higher in the oldest age group. **Conclusions:** The results indicate that physical and social interventions should be more relevant for older women with greater social isolation, as they may increase frailty indices and consequently the risk of hospitalization, institutionalization, and mortality.

## 1. Introduction

All human beings undergo an aging process, which is highly complex and variable, and therefore, determining key factors regarding quality of life and longevity is essential. Aging begins at birth and becomes more pronounced in the later stages of adulthood, making it a phenomenon not limited to older adults, but rather inherent to every individual [1], and it attracted the attention of researchers interested in understanding its multiple dimensions. Recently, particular focus has been placed on identifying the factors associated with frailty, with the goal of informing future interventions aimed at mitigating its signs and promoting healthier aging trajectories [2,3].

Frailty has been defined in multiple ways within existing literature, reflecting the complexity and evolving nature of the concept. Some definitions frame frailty primarily as a heightened susceptibility to both exogenous and endogenous stressors that exacerbate the likelihood of adverse health outcomes, including but not limited to falls, cognitive impairments, physical disabilities, hospitalization, and mortality [4,5]. However, frailty should not be equated as a synonym of comorbidity or disability, as each disease generates distinct care needs among older adults. For example, pre-frailty can be understood as a multifactorial, multidimensional, and dynamic syndrome that represents a transitional and potentially reversible risk state occurring prior to the onset of clinically manifest frailty. While it cannot be equated with disability, pre-frailty constitutes a ‘pre-disability’ condition insofar as it reflects subclinical impairments, often manifesting as fatigue, weakness, or other subtle functional declines. This may predispose individuals to adverse health outcomes, including the development of overt frailty, disability, hospitalization, and reduced quality of life [6,7]. Pre-frailty seems to exist along a continuum of frailty states and highlights the importance of early detection and intervention. Although consensus on a universally accepted definition is still lacking, current evidence supports its identification through measurable bio-psycho-social markers [5,8].

Conceptual definitions of frailty primarily focused solely on physical issues that affect older adults. However, frailty is not limited to physical capacity. Psychological and social dimensions are also essential for maintaining functional independence within the older adult community [8,9,10]. The physical dimension refers to the ability of older individuals to perform tasks that require physical effort, such as walking difficulties, reduced handgrip strength, or limitations in vision and hearing. The psychological dimension is defined as cognitive and emotional processing capacity, including memory impairment, feelings of nervousness or stress, and decreased levels of coping management when facing adversity. Lastly, the social dimension centers on interpersonal aspects of communication, such as lack of support from others, living alone, or feelings of loneliness. These three dimensions appear to be crucial, and high scores in some instruments in each are indicative of elevated levels of frailty in older adults [11,12].

There is a growing need to understand how frailty varies according to sociodemographic indicators within the aging population. While existing studies predominantly focused on identifying whether an individual is or is not frail [13,14], they often overlooked the broader context, such as sociodemographic characteristics in which frailty may occur. Specifically, factors such as marital status, age, diagnosed diseases, and medication use are commonly associated with aging and appear to influence the degree of frailty. As highlighted in the literature, lower socioeconomic position, reflected by indicators such as education, income, and occupational class, has been consistently associated with a higher prevalence of frailty [15,16,17]. These findings underscore the importance of considering a multidimensional framework when examining frailty, one that integrates both individual health characteristics and broader sociodemographic and clinical determinants.

Existing literature indicates that marital status may impact various health outcomes, including cardiovascular disease and mortality [12,18]. Social factors such as living alone, loneliness, and social isolation appear to have significant associations with the risk of frailty [19]. Consequently, it is expected that older adults who live in isolation or are widowed, divorced, or single may exhibit higher levels of frailty due to the lack of social interaction and support. Observational studies also found associations between advanced age and increased levels of frailty. However, as described earlier, frailty has often been considered solely in terms of its physical dimension, as demonstrated in studies linking age with physical fitness. Measurements of physical frailty have been conducted through agility and balance tests [14], body mass index [20], or handgrip strength [21,22].

Theoretically, frailty implies a higher risk of adverse outcomes due to the presence of comorbidities such as sarcopenia [23], malnutrition [24], and/or depression [3]. Therefore, it is reasonable to expect that older adults with multiple chronic diseases may exhibit higher levels of frailty compared to those without diagnosed illnesses. Indeed, chronic diseases (e.g., type II diabetes, hypertension, digestive issues, or musculoskeletal disorders) may influence physical frailty. However, the extent to which they affect psychological and social dimensions remains underexplored. It is possible that older adults with multiple disease may experience fear related to social interaction. For instance, an individual with a cardiorespiratory illness may fear walking to a social engagement, while someone with a neurological disease may experience mood or cognitive disturbances, thus also increasing their psychological frailty.

Regarding clinical determinants, recent studies suggest that the relationship between frailty and medication use is bidirectional: older adults who take more medications tend to exhibit higher levels of physical frailty, while frailty, often associated with the presence of chronic diseases, increases the likelihood or necessity of medication use [25]. Indeed, some studies investigated the association between the use of nonsteroidal anti-inflammatory drugs [26] and muscle relaxants [27] and an increased risk of physical frailty. However, frailty is not exclusive to the physical domain, making it equally important to examine the role of medication in psychological and social components. The literature highlights that certain medications may affect cognitive functioning, even those not prescribed specifically for dementia or cognitive impairment [28]. Among those medications are pain medications [29] and antidepressants [30] that could alter psychological and social frailty, respectively. Other medications prescribed for diseases such as hypertension may influence social and psychological components of frailty by affecting cognitive and emotional disposition [31].

This study focused exclusively on women. This decision was guided both by a growing body of literature demonstrating sex-specific differences in the presentation and progression of frailty. Research indicates that women exhibit higher prevalence rates of frailty than men, despite living longer and often reporting better self-perceived health [32,33]. These discrepancies are thought to stem from complex interactions between biological, psychological, and social factors, including differences in hormonal profiles, disease burden, and social roles throughout the life course [34,35]. Moreover, women are disproportionately affected by psychosocial stressors in older age, such as greater prevalence of widowhood, caregiving responsibilities, and lower socioeconomic status, which may further contribute to elevated frailty risk, particularly in the psychological and social domains [10,36]. Importantly, analyzing data solely from older women allows for greater specificity and depth in understanding the unique factors within this demographic, exploring the validity of the findings and their potential to inform specific interventions in geriatric care [37].

The aim of this study was to analyze levels of frailty, across physical, psychological, social, and overall dimensions, according to marital status, age, number of diagnosed diseases, and number of medications taken in older women. It is hypothesized that women who are married will present lower levels of frailty compared to those who are single or widowed. Conversely, it is expected that older women will exhibit higher levels of frailty than younger individuals. Additionally, it is hypothesized that older women with a higher number of diagnosed illnesses or greater medication use will present higher levels of frailty compared to those with fewer diagnoses or lower medication use, respectively.

## 2. Materials and Methods

### 2.1. Participants

A cross-sectional study was conducted at a single point in time to provide information on frailty indices among the older adult population. Participants in this study were older adult women living in the municipal community of Pombal (Portugal) who met specified inclusion criteria. Individuals were eligible to participate if they were women and aged 60 years or older. Participants were not included if they had a known diagnosis of cognitive impairment or any other neurological disease that could affect physical functioning, mobility, or balance, as these factors could potentially influence the frailty indexes. Furthermore, individuals with a recent history of falls, defined as two or more falls within the past six months, were also not included, as these factors could potentially influence the physical frailty indexes. All participants were required to be capable of independently providing informed consent.

The study included a total of 94 women participants (M = 71.33; SD = 7.88). In terms of age distribution, 44.7% (*n* = 42) were between 60 and 70 years old, 51.1% (*n* = 48) were aged 71 to 80 years, and 4.3% (*n* = 4) were between 81 and 90 years. Regarding educational attainment, the majority of participants (63.8%, *n* = 60) completed between 1 and 4 years of formal education. Additionally, 12.8% (*n* = 12) had 5 to 9 years of schooling, 6.4% (*n* = 6) completed 10 to 12 years, 4.3% (*n* = 4) attained more than 12 years of education, and 12.8% (*n* = 12) had no formal schooling. With respect to marital status, half of the sample (50.0%, *n* = 47) was indicated as being divorced, followed by 36.2% (*n* = 34) who were married, 10.6% (*n* = 10) who were widowed, and 3.2% (*n* = 3) who were single. Concerning clinical characteristics, the number of reported conditions ranged from zero to five, with the majority of participants experiencing between one and three diseases. Specifically, 14.9% (*n* = 14) reported no diagnosed diseases, whereas 20.2% (*n* = 19) had one disease, 17.0% (*n* = 16) had two, 27.7% (*n* = 26) had three, and 11.7% (*n* = 11) reported four diagnosed diseases. The most commonly reported conditions included hypertension (63%), hypercholesterolemia (53%), musculoskeletal disorders (43%), cardiovascular diseases (31%), and nervous system disorders (28%). Smaller proportions reported five diseases (*n* = 8; 8.5%). As for medication usage, 11.7% (*n* = 11) were not taking any medications, 23.4% (*n* = 22) were taking one to two medications, 34.0% (*n* = 32) were on three to four medications, 16.0% (*n* = 15) were taking five to six medications, and 14.9% (*n* = 14) were taking more than six medications.

### 2.2. Data Collection Procedures

Prior to the beginning of data collection, favorable ethical approval was obtained from the Ethics Committee of the Polytechnic Institute of Leiria (CE/IPLEIRIA/63/2024), and all procedures adhered to the ethical standards of the Declaration of Helsinki for research involving human participants. The data collection took place in July 2024. A convenience sampling method was used for data collection, as the researchers had an established protocol with an organization involved in the physical and social monitoring of older adults living in the community. The study was conducted in collaboration with a supporting staff, who assisted the researchers in identifying potential participants based on the inclusion criteria.

All interested participants received detailed information about the study and were given the opportunity to ask questions. Two experienced researchers explained the study’s purpose, procedures, and the instruments to be used, providing a clear demonstration of how to complete them. Written informed consent was obtained prior to participation. Data collection took place in private settings to ensure confidentiality, and participants were assured that their data would be used solely for research purposes, with no personal identifiable information collected, in line with ethical standards of anonymity. Participants were also informed of their right to withdraw from the study at any time without consequence.

### 2.3. Instruments

Initially, a sociodemographic questionnaire was administered, which included questions regarding age, marital status, educational level, diagnosed illnesses, and used medication. These data were previously reported in the sample characterization section.

In the second phase, participants completed a questionnaire to assess frailty using the Tilburg Frailty Indicator (TFI), which has been validated for the Portuguese population by Coelho et al. [38]. The TFI is a brief self-report instrument designed to assess frailty in community-dwelling older adults and consists of two subscales: Part A includes 10 items regarding determinants of frailty (e.g., age, sex, education, and income), and Part B includes 15 items focused solely on the components of frailty divided into three domains (physical, psychological, and social). For this study, only the items from Part B were used, avoiding overlapping information and providing a total frailty score as well as scores for each individual domain.

Considering this tool for assessing frailty, 11 items have dichotomous response categories (yes/no), while 4 items have three response options (yes/sometimes/no). However, all items are scored as either zero or one. The physical domain of the TFI includes eight items related to physical health, such as unexplained weight loss, difficulty walking, balance issues, hearing and vision problems, handgrip strength, and physical fatigue. The psychological domain comprises four items related to cognitive functioning, depressive and anxious symptoms, and coping mechanisms. The social domain includes three items focusing on living alone, social relationships, and social support. The TFI has been validated in several countries, including the Netherlands [21], Spain [26], Brazil [39], Poland [40], and Ethiopia [41], and demonstrated strong cross-cultural reliability [42].

### 2.4. Statistical Analysis

Descriptive and inferential analyses were conducted using IBM SPSS Statistics 29.0 (IBM, Armonk, NY, USA). Descriptive statistics were computed, including the median, interquartile range. Given the non-parametric nature of the data or the small sample sizes, all group comparisons were conducted using the Kruskal–Wallis H test. This test was employed to identify potential differences in frailty indices based on age, marital status, diagnosed diseases, and medication use groups. Statistical significance was determined at a *p*-value < 0.05. When significant differences were observed, pairwise post-hoc comparisons were performed using the Bonferroni correction to control for type I error.

Effect sizes for the Kruskal–Wallis test were estimated using epsilon squared (ε^2^), which provides an indication of the magnitude of the observed differences. The effect size interpretation followed common guidelines: small (ε^2^ ≈ 0.01), moderate (ε^2^ ≈ 0.06), and large (ε^2^ ≈ 0.14), consistent with recommendations for non-parametric analysis [43].

## 3. Results

The results of the descriptive and inferential analyses based on age group are presented in Table 1. The age categories used in this study were established a priori by the research team based on decade of age, aiming to reflect meaningful distinctions across stages of late adulthood commonly addressed in community-based ageing research, such as the sexagenarian (60 to 69 years), the septuagenarian (70 to 79 years), and octogenarian (80 to 89 years) [44]. With regard to frailty indices and the total frailty score, no statistically significant differences were found between age groups for the physical, social, or overall dimensions (*p* > 0.05). However, a statistically significant difference was observed in the psychological dimension (H = 6.15, df = 2, and *p* = 0.040), with post-hoc comparisons indicating that the 80–89 age group reported significantly higher psychological frailty scores compared to the 70–79 group.

The results of the descriptive and inferential analyses based on marital status are presented in Table 2. For physical frailty, the Kruskal–Wallis test yielded a statistically significant result (H = 9.01, *p* = 0.029). However, none of the pairwise post-hoc comparisons reached the Bonferroni-adjusted significance threshold. In the psychological frailty domain, significant group differences were observed (H = 10.34, *p* = 0.016). Post-hoc comparisons indicated that divorced individuals had significantly higher psychological frailty scores than married participants. The effect size was ε^2^ = 0.093, corresponding to a moderate effect. No statistically significant differences were found for the social frailty index (H = 3.12, *p* = 0.373). The total frailty index showed highly significant group differences (H = 13.66, *p* = 0.003). Post-hoc tests revealed that single individuals had significantly lower total frailty scores than divorced individuals and that married participants also scored significantly lower than their divorced counterparts. The corresponding effect size for total frailty was ε^2^ = 0.123, indicating a moderate to large effect.

The results of the descriptive and inferential analyses based on the number of diagnosed diseases are presented in Table 3. The results only reveal statistically significant differences in psychological frailty (*p* < 0.05). Participants with no diagnosed diseases reported significantly lower levels of psychological frailty compared to those with five diseases. The corresponding effect sizes indicated small to moderate effects (ε^2^ = 0.070).

The results of the descriptive and inferential analyses based on the number of medications used are summarized in Table 4. Significant differences in physical frailty were identified among the groups. Post-hoc pairwise comparisons revealed that participants not taking any medications had significantly lower physical frailty than those taking five to six and more than six medications. Additionally, the one to two medication group differed significantly from the five to six medication group, indicating lower physical frailty scores. No statistically significant differences were found in the psychological frailty dimension across medication groups. Similarly, social frailty showed no significant group differences since Bonferroni adjustment did not reach significance. Regarding total frailty scores, significant group differences were also observed. Participants not using medications exhibited lower total frailty compared to those taking five to six medications and more than six medications. The epsilon squared effect sizes were calculated as 0.141 for physical frailty and 0.128 for total frailty, indicating moderate to large effects of medication use on these frailty measures.

## 4. Discussion

The objective of this study was to examine frailty levels across physical, psychological, social, and global dimensions in relation to marital status, age, number of diagnosed diseases, and medication use in older adult women. In regard to age groups, a significant difference was observed in psychological frailty, with the oldest group (80–89 years) reporting higher scores than the 70–79 group. However, the statistically significant differences that emerged with respect to age groups did not differ significantly in physical, social, or total frailty. In contrast, widowed and divorced individuals exhibited higher physical and total frailty levels compared to married and single participants. Regarding the number of diagnosed diseases, participants with fewer diseases (one or two) showed significantly lower physical and total frailty compared to those with four diseases, while those with two diseases also had lower psychological frailty scores than those with four. These differences exhibited large effect sizes. Concerning medication use, participants taking fewer medications (none or one to two) had significantly lower physical and total frailty levels than those taking five to six or more than six medications, with large effect sizes observed. Overall, these findings suggest that marital status, clinical burden, and polypharmacy are important factors of frailty, particularly in the physical and global dimensions.

The results of the present study indicate that there were no statistically significant differences in physical, social, or total frailty indices across the three age groups analyzed. These findings suggest that frailty may be consistent with emerging perspectives, which emphasize the multidimensional and heterogeneous nature of the aging process [45,46]. The lack of significant differences between age groups in physical, social, and total frailty score may also reflect the increasing recognition that chronological age may be an imprecise indicator of frailty, and that frailty progression depends more on lifestyle, comorbidities, and psychosocial diseases than age per se [47]. Moreover, some authors proposed that cross-sectional studies, such as the present one, may fail to detect gradual or individualized patterns of decline that are better captured through longitudinal designs [48]. Thus, although the age brackets used in this study were established to represent distinct stages in older adulthood, the similar frailty profiles observed may underscore the importance of adopting comprehensive and individualized approaches when assessing frailty in aging populations. The significant increase in psychological frailty observed in the 80–89-year-old group compared to the 70–79-year-old cohort merits some attention. Psychological frailty, encompassing cognitive decline, emotional vulnerability, and reduced stress coping capacity, may exponentiate in the older stages of live, influencing overall frailty and health outcomes in older women [10,36]. Advancing age is associated with an increased risk of cognitive impairments, including mild cognitive decline and dementia, which directly contribute to psychological vulnerability [49]. Emotional challenges, such as increased prevalence of depression, anxiety, and loneliness, are also more frequent in advanced age, often due to losses in social networks, bereavement, and physical health deterioration [50]. These factors may exacerbate the psychological dimension of frailty, impairing resilience and coping resources. It is worth noting that the absence of statistically significant differences between age groups may also be attributed to the limited and uneven sample sizes across categories, particularly in the 80–89 years group, which likely reduced the statistical power to detect potential differences. Small sample sizes are known to increase the risk of type II error, limiting the ability to draw robust conclusions regarding age-related variations in frailty.

The present findings, derived from a women-only sample, underscore the significant association between marital status and frailty in later life, particularly in the psychological and total domains. Statistically significant group differences were observed for psychological frailty and total frailty, both indicating moderate to large effects. Divorced women consistently exhibited higher levels of frailty in these domains compared to their married and single counterparts. In particular, psychological frailty was significantly higher among divorced women than among married women, suggesting a possible emotional toll associated with marital dissolution. This aligns with previous research indicating that older women may experience intensified psychological vulnerability following divorce due to factors such as diminished social capital, heightened economic strain, and reduced access to informal caregiving networks [35,51]. Although physical frailty scores appeared higher in divorced and widowed groups, these differences did not reach statistical significance in post-hoc comparisons. Nevertheless, the elevated scores among widowed participants may still reflect the psychosocial consequences of spousal loss, including the potential erosion of daily emotional and instrumental support traditionally provided within marriage [52]. Importantly, no significant differences were observed in the social frailty domain. This suggests that marital status alone may not adequately capture the complexity of older women’s social support systems, which often extend beyond marital ties to include friendships, intergenerational relationships, and community engagement [53]. Of particular note is the finding that single women reported the lowest levels of total frailty. While the small sample size in this group warrants cautious interpretation, it may reflect protective psychosocial mechanisms associated with lifelong singlehood, such as greater autonomy, adaptive coping strategies, and the absence of caregiving-related stressors. Recent studies proposed that singlehood in older adulthood may offer certain health advantages, including increased lifestyle flexibility and more consistent self-care behaviors [51,53]. Taken together, these results contribute to a nuanced understanding of the interplay between marital status and frailty among older women. They highlight the need to consider gender-specific pathways, including caregiving histories, emotional resilience, and the structure of informal support networks, when interpreting health outcomes in aging populations. Future research should seek to replicate these findings in both sex samples and explore the distinct psychosocial and health implications of marital transitions among older men, who may experience these processes through different social, cultural, and behavioral lenses.

The current findings indicate a nuanced association between the number of diagnosed chronic diseases and frailty among older adult women. The number of reported conditions ranged from zero to five, with the majority of participants experiencing between one and three diseases. The most commonly reported conditions included hypertension, hypercholesterolemia, musculoskeletal disorders, cardiovascular diseases, and nervous system disorders, consistent with the epidemiological profile of multimorbidity in later life [3]. Less frequent conditions include neoplasms, hematological disorders, and renal or gastrointestinal diseases. While descriptive statistics suggested a general trend toward increasing frailty with a higher number of chronic diseases, inferential analyses revealed that statistically significant differences were limited to the psychological frailty domain. Post-hoc comparisons indicated that individuals with no diagnosed diseases exhibited significantly lower psychological frailty than those with four diagnosed diseases, with a small to moderate effect size. In contrast, differences across groups for physical, social, and total frailty indices did not reach statistical significance, despite observed tendencies suggesting higher frailty with increasing multimorbidity, which partially support the existing literature [3,6]. Notably, Villacampa-Fernández et al. [54] emphasize the importance of distinguishing between diseases as constitutive components of frailty versus comorbid contributors, a conceptual nuance that may explain the observed patterns. While physical and total frailty tended to rise with disease burden, the psychological domain was most sensitive to increases in the number of chronic conditions, suggesting that psychological vulnerability may be an early or particularly salient marker of disease-related decline in this population. Several mechanisms may underline the link between multimorbidity and psychological frailty, including increased emotional distress, reduced autonomy, and the cumulative burden of managing multiple illnesses [4,51]. Conditions such as cardiovascular disease and musculoskeletal pain, for instance, may not only impair physical function, but also contribute to increased depressive symptoms, anxiety, and a diminished sense of well-being [13]. However, the absence of significant group differences in the physical and social domains suggests that additional factors, such as functional adaptation, social support, and individual coping strategies, may moderate the frailty trajectory among older women with chronic illness. However, this needs to be tested before drawing conclusions. Finally, the limited number of participants with five or more diseases may constrain the statistical power to detect differences in the upper range of multimorbidity. As such, the findings should be interpreted cautiously and underscore the need for further research with larger and more diagnostically diverse samples. Future studies should also consider longitudinal designs to clarify the temporal sequencing and potential bidirectionality between multimorbidity and frailty.

The analysis of used medication in relation to frailty dimensions revealed significant associations, particularly in the physical and total frailty indices. Participants not taking any medications demonstrated substantially lower levels of physical frailty compared to those consuming five to six medications. Similarly, individuals in the one to two medication group exhibited significantly lower physical frailty than those in the five to six group. The effect size for physical frailty indicated a moderate to large effect, highlighting the clinical relevance of this relationship. In contrast, no statistically significant group differences emerged for psychological frailty, although descriptive trends suggested greater psychological vulnerability among those taking a higher number of medications. These patterns may imply that the adverse consequences of polypharmacy are more salient in the physical domain of frailty, though not entirely absent from psychological and social functioning. Total frailty scores also varied significantly across medication groups, with those not taking any medications presenting lower global frailty than individuals in the five to six and more than six medication categories. The effect size for total frailty further supports a moderate to large impact of medication burden on overall frailty. These findings align with previous research identifying polypharmacy as both a marker and a contributing factor to frailty, primarily due to its association with adverse drug reactions, medication interactions, and diminished physiological reserve [25,26,55,56]. Moreover, polypharmacy often results from the need to manage multimorbidity, yet it may paradoxically exacerbate frailty through cumulative effects on physical performance, cognitive functioning, and overall health status [30,31]. The current results underscore the need for proactive medication review and deprescribing strategies, particularly in geriatric populations. Integrating regular pharmacological assessments into routine care may be a critical step toward reducing frailty risk and improving health outcomes in older adults.

### Limitations and Future Directions

Despite the exploratory contributions of this study to the understanding of frailty in older adults, several limitations must be acknowledged. First, the cross-sectional design limits the ability to draw causal inferences. Consequently, future research should prioritize longitudinal or experimental study designs that allow for the development and evaluation of interventions targeting the physical, psychological, and social dimensions of frailty, dimensions which should be adapted based on the specific patterns identified in this study. Second, the assessment tool used, the Tilburg Frailty Indicator, is mostly based on self-reported dichotomous questions rather than objective physical, psychological, or social performance measures. Although the instrument is widely validated, it may not capture the full complexity of the frailty phenotype, particularly in its physical and psychological aspects. Therefore, future studies should consider incorporating complementary tools, such as physical performance tests or standardized psychological assessments, to provide a more comprehensive evaluation of frailty. Third, the relatively small sample size, particularly within certain sociodemographic subgroups (e.g., single or divorced individuals, advanced age groups), restricted the ability to conduct more granular comparisons within these categories. The sample also presented only older women, which may have influenced the results, as existing literature indicates that frailty can manifest differently by sex [21,44]. Finally, the study was conducted within a specific geographic region of Portugal, which may limit the generalizability of the findings to the wider elderly population at the national level. Nevertheless, the alignment of the results with theoretical and empirical literature supports the notion that the sample may retain a certain degree of representativeness.

Future research should explore additional variables not examined in the present study, such as social support networks, physical activity levels, nutritional status, and cognitive functioning, which may further elucidate the complex interplay of factors contributing to frailty. Expanding the scope of future investigations will be essential to advancing effective strategies for the prevention, early detection, and management of frailty in older adults.

## 5. Conclusions

The present study highlights the multifactorial character of frailty among older adult women, emphasizing the significant roles of social, clinical, and pharmacological determinants. Marital status was identified as a key factor, with married women exhibiting lower levels of physical and social frailty relative to their widowed peers, thereby suggesting that close interpersonal relationships may exert a protective influence against frailty progression. While the age factor alone did not demonstrate significant associations with frailty dimensions, both multimorbidity and polypharmacy were robustly associated with elevated physical and overall frailty levels. These findings underscore the interplay between chronic disease burden, medication use, and functional frailty in later life, reinforcing the necessity for comprehensive, multidisciplinary approaches including routine medication reviews to attenuate frailty risk. Frailty represents a vulnerable state within the aging continuum, often serving as a transitional phase between successful aging and functional dependence, with potential progression toward institutionalization. Importantly, given the reversibility of frailty in its initial stages, early detection and intervention are paramount. The current evidence advocates for the development and implementation of targeted preventive strategies aimed at delaying frailty onset based on sociodemographic and clinical determinants, thereby possibly promoting sustained autonomy, enhanced quality of life, and continued social engagement among older adults within their communities.

## Figures and Tables

**Table 1:** Descriptive and inferential analysis based on age.

Variables	60–69 Years (1)		70–79 Years (2)		80–89 Years (3)		*H*	df	*p*	Bonferroni-Adjusted Significance Level	Pairwise Post-Hoc Comparisons
	Mdn	IQR	Mdn	IQR	Mdn	IQR					
Physical	2.50	3.25	2.00	3.00	2.00	3.50	1.92	2	0.382	>0.05	-
Psychological	1.00	2.25	2.00	2.00	3.00	0.75	6.15	2	0.046	0.040	2 ≠ 3
Social	1.00	1.25	1.00	1.00	1.50	2.50	0.38	2	0.827	>0.05	-
Total	6.00	6.00	5.00	3.00	6.50	5.75	0.56	2	0.755	>0.05	-

Notes: Mdn = median; IQR = interquartile range; H = Kruskal–Wallis test; df = degrees of freedom; and *p* = significance level.

**Table 2:** Descriptive and inferential analysis based on marital status.

Variables	Single (1)		Married (2)		Divorced (3)		Widowed (4)		*H*	df	*p*	Bonferroni- Adjusted Significance Level	Pairwise Post-Hoc Comparisons
	Mdn	IQR	Mdn	IQR	Mdn	IQR	Mdn	IQR					
Physical	1.00	0.00	2.00	3.00	3.00	2.00	2.00	2.25	9.01	3	0.029	>0.05	-
Psychological	1.00	0.00	1.00	1.50	2.00	2.00	2.50	2.25	10.34	3	0.016	0.031	2 ≠ 3
Social	0.00	0.00	1.00	1.00	1.00	1.00	1.00	1.25	3.12	3	0.373	>0.05	-
Total	2.00	0.00	4.00	3.25	6.00	4.00	6.50	4.25	13.66	3	0.003	0.031; 0.033	1 ≠ 3; 2 ≠ 3

Notes: Mdn = median; IQR = interquartile range; H = Kruskal–Wallis test; df = degrees of freedom; and *p* = significance level.

**Table 3:** Descriptive and inferential analysis based on diagnosed diseases.

Variables	0 Diseases (1)		1 Disease (2)		2 Diseases (3)		3 Diseases (4)		4 Diseases (5)		5 Diseases (6)		*H*	df	*p*	Bonferroni- Adjusted Significance Level	Pairwise Post-Hoc Comparisons
	Mdn	IQR	Mdn	IQR	Mdn	IQR	Mdn	IQR	Mdn	IQR	Mdn	IQR					
Physical	2.50	2.50	2.00	1.00	3.50	3.75	3.00	2.25	3.00	2.00	2.00	2.50	10.71	5	0.05	>0.05	-
Psychological	1.00	0.25	1.00	3.00	2.00	2.00	2.00	2.00	3.00	2.00	2.50	2.00	12.16	5	0.03	0.036	1 ≠ 5
Social	0.50	1.00	1.00	1.00	2.00	1.00	1.00	1.00	1.00	1.00	1.00	0.75	10.44	5	0.06	>0.05	-
Total	3.50	4.00	4.00	4.00	6.50	5.75	7.00	4.00	6.00	4.00	5.50	2.50	13.44	5	0.02	>0.05	-

Notes: Mdn = median; IQR = interquartile range; H = Kruskal–Wallis test; df = degrees of freedom; and *p* = significance level.

**Table 4:** Descriptive and inferential analysis based on used medication.

Variables	0 Med (1)		1–2 Med (2)		3–4 Med (3)		5–6 Med (4)		>6 Med (5)		*H*	df	*p*	Bonferroni- Adjusted Significance Level	Pairwise Post-Hoc Comparisons
	Mdn	IQR	Mdn	IQR	Mdn	IQR	Mdn	IQR	Mdn	IQR					
Physical	1.00	2.00	2.00	2.50	2.00	3.00	4.00	3.00	4.00	3.00	19.969	4	<0.001	0.018; 0.002; 0.017	1 ≠ 4; 1 ≠ 5; 2 ≠ 4;
Psychological	1.00	3.00	1.50	2.00	1.00	1.75	3.00	2.00	2.00	2.00	7.010	4	0.135	>0.05	-
Social	1.00	1.00	2.00	1.00	1.00	0.00	2.00	1.00	2.00	1.00	10.395	4	0.034	>0.05	-
Total	3.00	5.00	6.00	4.00	5.00	2.75	8.00	3.00	7.50	3.25	18.504	4	<0.001	0.011; 0.007	1 ≠ 4; 1 ≠ 5

Notes: Mdn = median; Med = medications; Mdn = median; IQR = interquartile range; H = Kruskal–Wallis test; df = degrees of freedom; and *p* = significance level.

## Data Availability

The data utilized in this study were obtained under a specific license exclusively for the purposes of this research. The data supporting the findings of this study are publicly available upon reasonable request to AGEING@LAB–ANGES.
